# ACETABULAR COMPONENT ORIENTATION IN TOTAL HIP ARTHROPLASTY: THE ROLE OF ACETABULAR TRANSVERSE LIGAMENT

**DOI:** 10.1590/1413-785220162405158405

**Published:** 2016

**Authors:** MOHAMMED EL IDRISSI, ABDELHALIM ELIBRAHIMI, MOHAMMED SHIMI, ABDELMAJID ELMRINI

**Affiliations:** 1. Hassan II University Hospital, Orthopedics Department, Fez, Morocco.

**Keywords:** Hip, Arthroplasty, Hip dislocation

## Abstract

**Objective:**

The aim of our study is to present the benefit of using the transvers acetabular ligament for intraoperative determination of the anteversion of acetabular component.

**Methods:**

Twenty-one total hip arthroplasties were performed. The transverse acetabular ligament was identified and used as a guide to position the acetabular component.

**Results:**

The mean anteversion angle was 16.9. None of the patients studied sustained a postoperative dislocation during this short follow-up period.

**Conclusion:**

We conclude from this preliminary study that the transverse acetabular ligament can aid positioning of the acetabular component of a THR. It defines the version of the acetabular component without the need for external instrumentation, and is independent of the position of the patient. Level of Evidence IV; Prospective Study.

## INTRODUCTION

Acetabular cup positioning has a significant effect on the out- come of total hip arthroplasty (THA). It affects dislocation rate, component impingement, edge loading, accelerated bearing surface wear and osteolysis and/or loosening.^1^ In the course of surgery, the most important step is cup orientation. The position on the operating table, dislocation of the native hip and the use of retractors alter the pelvis and, thus, acetabular version.^2^ Several methods have been described in the literature and several devices are available from THA prosthesis manufacturers to help correct cup placement. Many authors have described the transverse acetabular ligament (TAL) as a patient-specific reference point in determining the correct acetabular anteversion. The aim of our study is to resent the benefit of using the transvers acetabular ligament for intraoperative determination of the anteversion of acetabular component.

## MATERIAL AND METHODS

A retrospective study was conducted between January 2013 and January 2015. It included 21 total hip arthroplasties performed by two senior surgeons. Ten patients were operated for hip osteoarthritis, eight for femoral neck fracture, and three for coxitis. Each patient had a postoperative anteroposterior (AP) radiograph of the pelvis, which was used to measure the abduction angle of the acetabular cup from lines drawn through the face of the cup and the inter-teardrop line. A standardized crosstable true lateral view of the hip was taken with the patient supine on the radiographic table with the contralateral lower extremity flexed as much as possible to eliminate lumbar lordosis and place the pelvis in a standard position. The anteversion angle of the acetabular component was measured according to the technique of Woo.^3^

### Surgical technique

**Figure1. Posterior:**
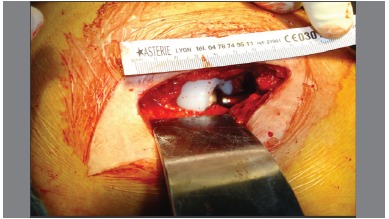
miniopen surgical approach.

**Figure 2:**
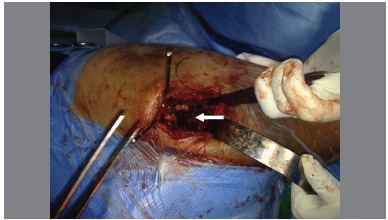
The TAL was carefully identified.

**Figure 3:**
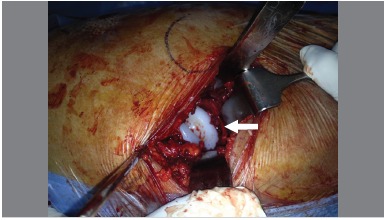
The TAL embraces the acetabular component.

Minopen posterior approach was used. (Figure 1) The piriformis tendon was preserved in all patients. As described by Archbold, retractors were used to obtain full exposure of the acetabulum. Inferiorly, the retractor was placed in a way that the transverse acetabular ligament remained superficial to it. The appearance of this ligament was graded using Archbold's classification.^4^ (Table 1) Once defined, (Figure 2) the transverse acetabular ligament was used as a guide to acetabular reaming. Then, the ligament embraced the final acetabular reamer and thereby, the acetabular component. (Figure 3)

**Table 1:** Intraoperative classification of the acetabular transverse ligament (ATL) according to Archbold^4^.

Grade of ATL	Appearance of ATL visualized during THA
1	Normal quality ATL visible at the acetabulum exposure
2	ATL covered by soft tissue which has to be removed to expose the ligament
3	ATL covered by osteophytes which have to be removed to expose the ligament
4	Ligament not identified, even after proper removal of osteophytes or soft tissue

The Hospital Ethics Committee approved the study, and all patients signed a Free and Informed Consent form before the surgery.

## RESULTS

The transverse acetabular ligament was identified and exposed in all cases. Radiological results are shown in Table 2. The mean anteversion angle was 16.9°. None of the patients studied showed a postoperative dislocation during the short follow-up period. 

**Table 2:** Radiological results.

Patient	Diagnosis	Anteversion angle (degrees)	Abduction angle (degrees)
P1	hip osteoarthritis	11	38
P2	hip osteoarthritis	15	30
P3	hip osteoarthritis	9	35
P4	hip osteoarthritis	8	41
P5	hip osteoarthritis	28	40
P6	hip osteoarthritis	18	43
P7	hip osteoarthritis	13	48
P8	hip osteoarthritis	15	37
P9	hip osteoarthritis	27	35
P10	hip osteoarthritis	16	45
P11	Fracture of the femoral neck	21	33
P12	Fracture of the femoral neck	15	32
P13	Fracture of the femoral neck	25	39
P14	Fracture of the femoral neck	24	32
P15	Fracture of the femoral neck	18	42
P16	Fracture of the femoral neck	20	44
P17	Fracture of the femoral neck	21	43
P18	Fracture of the femoral neck	16	48
P19	Coxitis	16	46
P20	Coxitis	18	45
P21	Coxitis	16	41
Mean (SD)		16.9 (±5)	39.85 (±5)

## DISCUSSION

Positioning of the acetabular component in THA has been shown to affect the range of motion of the hip, wear of the polyethylene, and the rate of pelvic osteolysis, being of paramount importance in minimizing the risk of dislocation.^5^ ^-^ ^8^ Unlike inclination, which according to most authors should be approximately 45°^6^ ^,^ ^9^ ^-^ ^11^ there is no consensus about how much anteversion is needed for the acetabular cup. Murray^12^ has defined three types of cup anteversion: anatomical, operative and radiographic. Anteversion values may differ depending on the plane of reference, and Murray has developed conversion nomograms for these three definitions. Lewinnek et al.^7^ described a 'safe zone' for acetabular anteversion as being 15 ± 10°, using a posterolateral approach. In the course of surgery, the most important step is cup orientation. The position on the operating table, the dislocation of the native hip and the use of retractors may alter the pelvis and, thus, the acetabular version.^12^ Several methods have been described in the literature and several devices are available from THA prosthesis manufacturers to help cup correct placement.^13^ ^-^ ^15^ Many studies have recommended the use of the triangular acetabular ligament (TAL) for acetabular component positioning.^4^ ^,^ ^16^ ^-^ ^19^ Although navigation technology has been shown to significantly improve precision during insertion of the acetabular component, the additional cost and increased duration of surgery has prevented the widespread uptake of these techniques.^20^ ^,^ ^21^ Furthermore, considering the variation in the patients' anatomy and their functional pelvic tilt, one might question the necessity of achieving standard acetabular implant orientation in every patient. In order to achieve a reliable and patient-specific alignment, there is a need for technical aids and tools base on reproducible anatomical landmarks.^22^

Archbold et al.^4^ have introduced the use of the TAL as a patient-specific reference point in determining the correct acetabular anteversion. With a large sample size, the TAL was easily identified in most cases and a very low rate of dislocation was observed. These authors present an easily reproducible and valid technique which is of widespread use today.

In order to assess the influence of acetabular component alignment relative to TAL and the posterior labrum as soft-tissue landmarks, Kalteis et al.^23^ ^,^ ^24^ measured the orientation of the native acetabular plane as defined by the transverse acetabular ligament and the posterior labrum intra-operatively, using computer-assisted navigation. They suggest that this alignment can reduce malpositioning of the acetabular component in total hip replacement according to the traditional safe zones. However, there is no improvement in range of movement and no decrease in impingement in an anatomical orientation when the acetabular component is aligned to the TAL and the posterior labrum, as compared to a standard orientation at 45° of abduction and 15° of anteversion. Viste et al.^25^ suggested that that cup orientation should be specific for each patient and not a universal standard, as suggested by Lewinnek et al.^7^ Indeed, anteversion is not a static parameter, but a specific dynamic value for each individual. According to Archbold et al.,^4^ this technique may not be applicable where a significant acetabular structural abnormality is present, as in severe dysplasia or following a pelvic fracture.

In our study, we had no case of hip dislocation. The TAL was easily identified in all patients. However, a larger and comparative study is needed to confirm this results.

## CONCLUSION

We conclude from this preliminary study that the transverse acetabular ligament can assist to position the acetabular component in THR. It defines the degree of version of the acetabular component without the need for external instrumentation, and it is independent of the patient's position.
